# Supplementary material for: Optimizing tuberculosis screening for immigrants in southern New Brunswick: A pilot study protocol
Source: PLoS One. 2022 Nov 4;17(11):e0277255. doi: 10.1371/journal.pone.0277255 (PMC9635694; doi:10.1371/journal.pone.0277255)
Supplement: S1 Appendix — (PDF) [file pone.0277255.s001.pdf]

Appendix 7a

Informed Consent Form for Participants

Title of Project: Optimizing Tuberculosis Screening for Newcomers in Southern New Brunswick: A Pilot Study

Introduction

We are inviting adults 19 years of age and older to participate in a study on screening for latent (dormant) tuberculosis infection. The purpose of this study is to gather information to assess the feasibility of implementing a latent tuberculosis infection screening program for newcomers from countries with many cases of tuberculosis.

If you choose to participate, you will be asked to provide about 4 ml of blood for testing latent tuberculosis infection after which it will be disposed of using the Saint John Regional Hospital protocols (i.e, it will not be stored or used in any other way). You will also be asked to complete an online survey for approximately 10 minutes about your experience in participating in the study.

Your participation is voluntary, and you may withdraw from the study at any time without any impact on the care or services or anything else you may associate with the newcomer services/centres/medical, etc. There is no cost for participating in the study. Participants will receive up to $40 in e-gift cards for their time, i.e., $20 after blood collection and another $20 after completing the online survey.

Potential benefits

The potential benefits from participating in this study may include the reassurance that you do not have latent tuberculosis infection (if the test is negative). If the test is positive and you get treated, you will benefit by considerably reducing the chances of suffering from tuberculosis disease, thereby keeping your family and friends safe.

Potential risks:

There is a minimal risk of discomfort, bruising, or infection at that site during blood collection, similar to giving an amount of blood for other medical tests.

Privacy and confidentiality

Protecting your privacy is an important part of this study. Information gathered from this study is strictly confidential, and your information will be anonymized. All electronic data will be stored on a secure drive at the Department of Nursing & Health Sciences at the University of New Brunswick for 5 years to allow for the dissemination of information, after which it will be destroyed. The data will only be accessed by members of the study team.

QUESTIONS

If you have questions after you read this form, ask the research team member assessing you. You should not sign this form or provide verbal consent until you are sure that you understand the study.

This project has been reviewed by the Research Ethics Board of the University of New Brunswick and is on file as REB File #033-2021, and the Horizon Health Network Research Ethics Board and poses minimal risk. In the event that injury, illness or disability results and you believe that it is related to your participation in this study, or if you have any questions about your rights as a research participant, you may contact Dr. Beth Keyes, Chair of the Research Ethics Board at the UNBSJ by phone [506-648-5994] or by email [REB@unb.ca] or Regional Director of Ethics Services, Horizon Health Network Research Ethics Board by phone [506) 648-6094] or by email at [REBOffice@HorizonNB.ca].

PARTICIPANTS STATEMENT

I have read the information about this study and have had the opportunity to discuss this study and my questions have been answered to my satisfaction. I acknowledge that I have been informed that my participation is voluntary and that the data I provide will remain confidential. I hereby consent to take part in this study.

Name of participant ______________________________

Signature of participant ____________________ Date ____________________

OR

Obtained verbal consent of participant ☐ Date ____________________

Please note that by consenting to participating in this study, you have not waived any rights to legal recourse in the event of research-related harm.

STATEMENT BY PERSON PROVIDING INFORMATION ON STUDY

I have explained to the above participant the nature, requirements and the purpose of the study, potential benefits, and possible risks associated with participation in this study. I have answered any questions that have been raised. I believe that the participant understands the implications and the voluntary nature of the study.

Researcher Signature: ______________________ Date: ____________

Research Team:

Dr. Duncan Webster, Division of Infectious Diseases, Department of Medicine, Saint John Regional Hospital, duncan.webster@horizonnb.ca

Dr. Isdore Chola Shamputa, Department of Nursing & Health Sciences, University of New Brunswick Saint John, chola.shamputa@unb.ca

Dr. Kimberly Barker, Medical Officer of Health, South Region, New Brunswick Department of Health, Kimberley.Barker@gnb.ca

Dr. Duyen Nguyen, Regional Senior Program Advisor, Government of New Brunswick, duyen.nguyen@gnb.ca

Formulaire de consentement éclairé pour les participants

Titre du projet: Optimisation du dépistage de la tuberculose chez les nouveaux arrivants dans le sud du Nouveau-Brunswick : Une étude pilote

Introduction

Nous invitons les adultes âgés de 19 ans et plus à participer à une étude sur le dépistage de l'infection tuberculeuse latente (dormante). L'objectif de cette étude est de recueillir des informations pour évaluer la faisabilité d'un programme de dépistage de l'infection tuberculeuse latente chez les nouveaux arrivants originaires de pays où les cas de tuberculose sont nombreux.

Si vous choisissez de participer, on vous demandera de fournir environ 4 ml de sang pour le dépistage de l'infection tuberculeuse latente, après quoi l'échantillon sera éliminé selon les protocoles de l'Hôpital régional de Saint John (c'est-à-dire qu'il ne sera ni stocké ni utilisé d'une autre manière). Nous vous demanderons également de répondre à un sondage en ligne d'environ 10 minutes sur votre expérience de participation à l'étude.

> Votre participation est volontaire et vous pouvez vous retirer de l'étude à tout moment sans affecter les soins ou les services ou tout ce qui se rapporte aux services/centres/médecine pour nouveaux arrivants. La participation à l'étude ne coûte rien. Les participants recevront jusqu'à 40 $ en cartes-cadeaux numériques pour le temps qu'ils auront consacré à l'étude, c'est-à-dire 20 $ après la collecte de sang et 20 $ après avoir répondu à l'enquête en ligne.

Avantages potentiels

Les avantages potentiels de la participation à cette étude peuvent inclure l'assurance que vous n'avez pas d'infection tuberculeuse latente (si le test est négatif). Si le test est positif, et que vous êtes traité, vous bénéficierez d'une réduction considérable des risques de souffrir de la tuberculose, vous permettant de protéger votre famille et vos amis.

Risques potentiels

Il existe un risque minime d'inconfort, d'ecchymoses ou d'infection au site de prélèvement du sang, comme pour n'importe queldon de sang pour un examen médical

Vie privée et confidentialité

La protection de votre vie privée est un aspect important de cette étude. Les renseignements recueillis dans le cadre de cette étude sont strictement confidentiels, et vos renseignements seront anonymes. Toutes les données électroniques seront sauvegardéessur un disque dur sécurisé au Département des sciences infirmières et de la santé de l'Université du Nouveau-Brunswick pendant 5 ans, afin de permettre la diffusion de l'information, après quoi elles seront détruites. Seuls les membres de l'équipe de l'étude auront accès aux données.

QUESTIONS

Si vous avez des questions après avoir lu ce formulaire, nous vous prions de les adresser au membre de l'équipe de recherche qui effectue votre évaluation. Nous vous recommandons de ne

pas signer ce formulaire ou donner votre consentement verbal avant d'être sûr de bien comprendre l'étude.

Ce projet a été évalué par le conseil déontologique de la recherche de l'Université du Nouveau-Brunswick et figure dans le dossier REB no 033-2021, et le comité déontologique de la recherche du réseau de santé Horizon et il pose un risque minimal. En cas de blessure, de maladie ou d'invalidité que vous croyez être liée à votre participation à cette étude, ou si vous avez des questions sur vos droits en tant que participant.e à la recherche, veuillez communiquer avec la Dre Beth Keyes, Présidente du conseil déontologique de la recherche de l'UNBSJ par téléphone [506-648-5994] ou par courriel [REB@unb.ca] ou la Direction régionale des services déontologiques, du conseil déontologique de la recherche du réseau de santé Horizon par téléphone [506) 648-6094] ou par courriel [REBOffice@HorizonNB.ca].

DÉCLARATION DES PARTICIPANT.E.S

J'ai bien lu les informations sur cette étude, j'ai eu l'occasion de discuter de cette étude etj'ai obtenu des réponses satisfaisantes à mes questions. Je reconnais avoir été informé que ma participation est volontaire et que les données que je fournis resteront confidentielles. Je consens par la présente à participer à cette étude.

Nom du participant.e ____

Signature du participant.e ______________________ Date ________________________

Ou

Obtention du consentement verbal du participant.e ☐ Date ________________________

Veuillez noter qu'en consentant à participer à cette étude vous ne renoncez à aucun droit de recours juridique en cas de préjudice lié à la recherche.

DÉCLARATION DE LA PERSONNE FOURNISSANT L'INFORMATION SUR L'ÉTUDE

J'ai expliqué au participant.e dont le nom figure ci-dessus la nature, les exigences et l'objectif de cette étude, les avantages potentiels et les risques possibles associés à sa participation à cette étude. J'ai répondu à toutes les questions qui m'ont été posées. Je crois que le participant.e comprend les implications et la nature volontaire de l'étude.

Signature du chercheur/ de la chercheuse ____________________ Date: ______________

L'équipe de recherche :

Dr Duncan Webster, Division des maladies infectieuses auDépartement de la médecine, Hôpital régional de Saint John, duncan.webster@horizonnb.ca

Dr Isdore Chola Shamputa, Département des soins infirmiers et des sciences de la santé à l'Université du Nouveau-Brunswick Saint John, chola.shamputa@unb.ca

Dre Kimberly Barker, Médecin-conseil en santé publique, Région Sud, Nouveau-Brunswick au Ministère de la santé Nouveau Brunswick ,Kimberley.Barker@gnb.ca

Dre Duyen Nguyen, Conseillère principale en programmes régionaux, au Gouvernement du Nouveau-Brunswick, duyen.nguyen@gnb.ca

Formulario de consentimiento informado para participantes

Título de Proyecto: Optimizando la detección de tuberculosis para recién llegados en el Sur de New Brunswick: Prueba piloto

Introducción

Invitamos a adultos de 19 años de edad o más a participar en un estudio sobre la detección de infecciones latentes de tuberculosis (inactivas). El propósito de la investigación es recoger información para evaluar la factabilidad de implentar un programa de detección de infecciones latentes de tuberculosis para recién llegados a Canadá procedentes de países con altos casos de tuberculosis.

Si usted decide participar, se le solicitará dar aproximadamente 4 ml de sangre para hacer la prueba de infección latente de tuberculosis, ésta será desechada posteriormente utilizando los protocolos del Hospital Regional de Saint John (Es decir que, la sangre no será utilizada ni almacenada de ninguna otra forma). Se le pedirá llenar una encuesta en línea que le tomará aproximadamente 10 minutos, sobre la experiencia de haber participado en esta investigación.

Su participación es voluntaria y puede retirarse del estudio en cualquier momento, sin afectar su acceso a los servicios de los centros de recién llegados, servicios médicos, etc. No hay costo por participar en la investigación. Los participantes puede recibir hasta $40 en tarjetas de regalo digitales por su tiempo. Por ejemplo: $20 después de extraer la sangre y $20 después de llenar la encuesta en línea.

Beneficios potenciales

Los beneficios potenciales de participar en ésta investigación pueden incluir: la certeza de que usted no tiene una infección latente de tuberculosis (si la prueba resulta negativa). Si la prueba resulta positiva y recibe tratamiento, usted se beneficiará al reducir considerablemente las posibilidades de sufrir de tuberculosis, y de esa forma mantener seguros a su familia y sus amigos.

Riesgos potenciales:

Hay un riesgo mínimo de malestar, hematomas, o infección en el sitio en el que se le extrajo la sangre, similar a lo que ocurre al dar sangre por otras pruebas médicas.

Privacidad y confidencialidad

Proteger a su privacidad es una parte importante de esta investigación. La información que se recoja de ésta investigación es estrictamente confidencial y anónima. Todos los datos electrónicos serán almacenados en una unidad segura en el Departamento de Enfermería y Ciencias de Salud de la Universidad de New Brunswick por 5 años, para permitir la difusión de la información después de lo cual, dicha información será destruida. Únicamente los miembros del equipo de la investigación tendrán acceso a dichos datos.

PREGUNTAS

Si tiene preguntas tras leer éste formulario, consulte al miembro de equipo de investigación que lo evaluó. No debería firmar este formulario o dar consentimiento verbal hasta que esté seguro que comprende la investigación.

Este proyecto ha sido revisado por la Mesa de Investigación Ética de la Universidad de New Brunswick y está archivado como REB File #033-2021, y la Mesa de Investigación Ética de la Red de Salud de Horizon e implica riesgos mínimos. En el caso que resultara alguna lesión, enfermedad o discapacidad y usted cree que está relacioanda con su participación en ésta investigación, o si usted tiene preguntas sobre sus derechos com participante en esta investigación, debe contactar a Dr. Beth Keyes, Presidenta de la Mesa de Investigación Ética de la Universidad de New Brunswick, Saint John, por teléfono al (506)-648-5994 o por email a [REB@unb.ca], o al Director Regional de Servicios Éticos de la Mesa de Investigación Ética de la Red de Salud de Horizon, por teléfono al (506) 648-6094 o por correo [REBOffice@HorizonNB.ca].

DECLARACIÓN DE LOS PARTICIPANTES

He leído la información acerca de la investigación, he tenido la oportunidad de discutir sobre este estudio y mis inquietudes han sido respondidas a un nivel satisfactorio. Recognozco que he sido informado que mi participación es voluntaria y los datos que proporcionaré serán confidenciales. Doy mi consentimiento para participar en esta investigación.

Nombre del participante ________________________________

Firma del participante ____________________ Fecha ____________________

O

Consentimiento verbal del participante ☐ Fecha ____________________

Por favor tenga en cuenta que por el acto de dar su consentimiento en esta investigación, no ha renunciado a ningún derecho de utilizar algún recurso legal en caso de algún daño relacionado con esta investigación.

DECLARACIÓN DE LA PERSONA QUE FACILITA LA INFORMACIÓN SOBRE ESTA INVESTIGACIÓN

He explicado a la persona indicada arriba, la naturaleza, los requisitos, y el propósito de la investigación, beneficios potenciales y riegos posibles relacionado con su participación. He respondido a cualquier duda del participante. Considero que el participante entiende las implicaciones de la investigación y que su participación en la misma es voluntaria.

Firma de investigador: ____________________ Fecha: ____________

Equipo de investigación:

Dr. Duncan Webster, División de Enfermedades Infecciosas, Departamento de Medicina, Hospital Regional de Saint John, duncan.webster@horizonnb.ca

Dr. Isdore Chola Shamputa, Departamento de Enfermería y Ciencias de Salud, Universidad de New Brunswick, Saint John, chola.shamputa@unb.ca

Dra. Kimberly Barker, Oficial Medico de Salud, Región Sur, Departamento de Salud de New Brunswick, Kimberley.Barker@gnb.ca

Dr. Duyen Nguyen, Asesor Principal del Programa Regional, Gobierno de New Brunswick, duyen.nguyen@gnb.ca

Foomka Oggolaanshaha Xog -ogaalka ah ee Ka -qaybgalayaasha

Cinwaanka Mashruuca: Hagaajinta Baaritaanka Qaaxada ee Dadka Soogolotiga Cusub ee Koonfurta New Brunswick: Daraasad Duuliye

Hordhac
Waxaan ku martiqaadeynaa dadka qaangaarka ah ee jira 19 sano iyo ka weyn inay ka qeybgalaan daraasad ku saabsan baaritaanka cudurka qaaxada qarsoon (hurda). Ujeeddada daraasaddan ayaa ah in la ururiyo macluumaad si loo qiimeeyo suurtogalnimada hirgelinta barnaamijka baaritaanka cudurka qaaxada ee qarsoon ee loogu talagalay dadka cusub ee ka yimid dalal badan oo qaba cudurka qaaxada.

Haddii aad doorato inaad kaqaybqaadato, waxaa lagaa codsan doonaa inaad bixiso qiyaastii 4 ml oo dhiig ah si loo baaro caabuqa qaaxada ee qarsoon ka dib markaas ayaa laga takhalusi doonaa adeegsiga borotokoollada Isbitaalka Gobolka Saint John (tusaale ahaan, lama kaydin doono ama si kale looma isticmaali doono). Waxa kale oo lagaa codsan doonaa inaad dhammaystirto sahan onlayn ah qiyaastii 10 daqiiqo oo ku saabsan waayo-aragnimadaada ka-qaybgalka daraasadda.

Ka-qaybgalkaagu waa ikhtiyaari, waxaadna ka bixi kartaa daraasadda wakhti kasta adiga oo aan wax saamayn ah ku yeelanayn daryeelka ama adeegyada ama wax kasta oo kale oo aad la xiriiri karto adeegyada ku soogolotiga-cusub/xarumaha / caafimaadka, iwm. Ma jirto wax kharash ah oo kaqaybqaadashada daraasadda. Ka-qaybgalayaashu waxay heli doonaan ilaa $40 kaararka elektaroonigga ah waqtigooda, tusaale ahaan, $20 ka dib ururinta dhiigga iyo $20 kale ka dib markay dhammaystiraan sahanka internetka.

Faa'iidooyinka iman kara
Faa'iidooyinka ka iman kara ka -qaybgalka daraasaddan waxaa ka mid noqon kara xaqiijinta inaadan qabin cudurka qaaxada ee qarsoon (haddii baaritaanka uu yahay mid taban).Haddii baaritaanka lagaa helo oo lagu daaweeyo, waxaad ka faa'iideysan doontaa adigoo si weyn hoos ugu dhigaya fursadaha uu kugu dhici karo cudurka qaaxada.

Khataraha iman kara:
Waxaa jira halis yar oo ah raaxo -darro, nabar, ama caabuq goobtaas inta lagu jiro ururinta dhiigga, oo la mid ah in la siiyo qaddar dhiig ah baaritaanno caafimaad oo kale.

Asturnaanta iyo qarsoodinimada
Ilaalinta asturnaantaada ayaa qayb muhiim ah ka ah daraasaddan. Macluumaadka laga soo ururiyey daraasaddan si adag ayaa qarsoodi ah, macluumaadkaagana waa la qarinayaa. Dhammaan xogta elektiroonigga ah waxaa lagu kaydin doonaa waddo aamin ah Waaxda Sayniska Caafimaadka Kalkaalinta ee Jaamacadda New Brunswick muddo 5 sano ah si loogu oggolaado faafinta macluumaadka, ka dib waa la burburin doonaa. Xogta waxaa heli doona oo kaliya xubnaha kooxda daraasadda.

SU'AALO
Haddii aad qabtid su'aalo ka dib markaad akhrido foomkan, weydii xubinta kooxda cilmi - baarista oo ku qiimeyneysa. Waa inaadan saxiixin foomkan ama aadan bixin oggolaansho afka ah ilaa aad hubto inaad fahamtay daraasadda.

Mashruucan waxaa dib-u-eegay Guddiga Anshaxa Cilmi-baarista ee Jaamacadda New Brunswick wuxuuna ku jiraa faylka REB File #033-2021, iyo Guddiga Anshaxa Cilmi-baarista Shabakadda Caafimaadka Horizon wuxuuna leeyahay halis yar. Haddii ay dhacdo dhaawac, jirro ama naafonimo oo aad rumeysan tahay inay la xiriirto ka -qaybgalkaaga daraasaddan, ama haddii aad wax su'aalo ah ka qabtid xuquuqdaada ka -qaybgale cilmi -baaris, waxaad la xiriiri kartaa Dr. Beth Keyes, Guddoomiyaha Guddiga Anshaxa Cilmi -baarista ee UNBSJ taleefan [506-648-5994] ama iimayl ahaan [REB@unb.ca] ama Agaasimaha Gobolka ee Adeegyada Anshaxa, Guddida Anshaxa Cilmi -baadhista Shabakadda Caafimaadka ee Horizon telefoon ahaan [506) 648-6094]ama iimayl ahaan [REBOffice@HorizonNB.ca].

BAYAANKA KA QAYBGALAYAASHA
Waxaan akhriyay macluumaadka ku saabsan daraasaddan waxaanan fursad u helay inaan ka doodo daraasaddan su'aalahaygiina waa laga jawaabay anigoo ku qanacsan. Waxaan qirayaa in la igu wargaliyay in kaqaybgalkaygu yahay ikhtiyaari iyo in xogta aan bixiyo ay ahaan doonto qarsoodi. Waxaan halkan ku oggolaaday inaan ka qaybqaato daraasaddan.

Magaca ka qaybgalaha ______________________________

Saxiixa ka qaybqaataha ____________________ Taariikhda ____________________

AMA

La helay oggolaansho afka ah ee ka qaybgalaha ☐ Taariikhda ____________________

Fadlan ogow adiga oo oggolaaday ka-qaybgalka daraasaddan, ma aadan ka tegin wax xuquuq ah oo ku aaddan dib-u-helidda sharciga haddii ay dhacdo waxyeello la xiriirta cilmi-baarista.

BAYAANKA QOFKA BIXINAYA MACLUUMAADKA WAXBARASHADA
Waxaan u sharxay ka -qaybgalaha kor ku xusan dabeecadda, shuruudaha iyo ujeeddada daraasadda, faa'iidooyinka iman kara, iyo khataraha suurtagalka ah ee la xiriira ka -qaybgalka daraasaddan. Waxaan ka jawaabay su'aalo kasta oo la soo qaaday. Waxaan aaminsanahay in kaqaybgalaha uu fahmay saamaynta iyo dabeecadda ikhtiyaariga ah ee daraasadda.

Saxiixa Cilmi-baarista: ______________________ Taariikhda: ____________

Kooxda Cilmi-baarista:

Dr. Duncan Webster, Qeybta Cudurada faafa, Waaxda Daawada, Isbitaalka Gobolka Saint John, duncan.webster@horizonnb.ca

Dr. Isdore Chola Shamputa, Department of Nursing & Health Sciences, University of New Brunswick Saint John, chola.shamputa@unb.ca

Dr. Kimberly Barker, Medical Officer of Health, South Region, New Brunswick Department of Health,
Kimberley.Barker@gnb.ca

Dr. Duyen Nguyen, Regional Senior Program Advisor, Government of New Brunswick,
duyen.nguyen@gnb.ca

الملحق 7أ

نموذج الموافقة المسبقة للمشاركين

عنوان المشروع: دراسة تجريبية لتحسين فحص مرض السل للقادمين الجدد في جنوب نيوبرونزويك.

مقدمة:

نحن ندعو البالغين من العمر 19 عاماً أو أكثر للمشاركة في دراسة لفحص مرض السل (الكامن). والغرض من هذه الدراسة هو جمع معلومات لتقييم جدوى تنفيذ برنامج فحص مرض السل الكامن للقادمين الجدد من بلاد تعاني من العديد من حالات السل.

إذا اخترت المشاركة في هذه الدراسة، سوف يُطلب منك تقديم حوالي 4 مل من الدم لفحص عدوى السل الكامنة ، وبعد ذلك سيتم التخلص منها وفقاً لبروتوكولات مشفى سانت جون (Saint John Regional Hospital)على سبيل المثال ، لن يتم تخزينها أو استخدامها بأي طريقة أخرى. و سيُطلب منكم أيضاً المشاركة في دراسة استطلاع رأي على الإنترنت لمدة 10 دقائق تقريباً عن تجربتكم في هذه الدراسة.

مشاركتك في هذه الدراسة تطوعية ، ويمكنك الإنسحاب في أي وقت دون أي تأثير على الرعاية أو الخدمات أو أي شيء آخر قد تربطه بأي خدمات/ مراكز طبية للوافدين الجدد ، إلخ. لا توجد تكلفة للمشاركة في الدراسة و سيتلقى المشاركون بطاقات هدايا إلكترونية قيمتها 40 $ تقديراً للمشاركة في وقتهم. سوف تتلقى 20$ بعد سحب عينة الدم و 20$ أخرى بعد الانتهاء من استطلاع الرأى الالكتروني.

الفوائد المحتملة:

و تشتمل الفوائد المحتملة من المشاركة في هذه الدراسة هي الطمأنينة أنك لست مصاب بمرض السل الكامن Latent Tuberculosis (إذا كانت نتيجة الإختبار سلبية). وإذا كانت النتيجة إيجابية وتلقيت العلاج اللازم سوف ينعكس ذلك بتقليل فرص الإصابة بمرض السل إلى حد كبير وبالتالى الحفاظ على سلامة أسرتك وأصدقائك.

المخاطر المحتملة:

هناك احتمال ضئيل جداً من الإحساس بعدم الراحة أوالكدمات الخفيفة مكان أخد عينة الدم، أو العدوى أثناء جمع الدم ، على غرار إعطاء عينة من الدم أثناء إجراء أي فحوصات طبية أخرى.

الخصوصية والسرية:

حماية خصوصيتك جزء مهم من الدراسة. سوف يتم الحفاظ على سرية هذه المعلومات بشكل صارم وسرى للغاية، وسوف تُخزن جميع البيانات الإلكترونية في مكان آمن في قسم التمريض والعلوم الصحية في جامعة نيوبرونزويك لمدة 5 سنوات للسماح بنشر المعلومات ثم تدميرها. هذه المعلومات سوف يتم مداولتها بين فريق البحث العلمي لهذه الدراسة فقط.

أسئلة:

إذا كانت لديك أسئلة بعد قراءة هذا النموذج ، فاطلب من عضو فريق البحث مساعدتك. يجب ألا توقع على هذا النموذج أو تقدم موافقة شفهية قبل التأكد من فهمك التام للدراسة.

تمت مراجعة هذا المشروع من قبل مجلس أخلاقيات البحث في جامعة نيو برونزويك وهو موجود في ملف REB File # 033-2021 ومجلس أخلاقيات أبحاثNetwork Health Horizon .تتضمن الدراسة الحد الأدنى من الخطر

في حالة حدوث إصابة أو مرض أو إعاقة وتعتقد أنها مرتبطة بمشاركتك في هذه الدراسة ، أو إذا كان لديك أي أسئلة حول حقوقك كمشارك في البحث ، يمكنك االاتصال بالدكتور بيث كييز" Beth Keyes "رئيس قسم البحث ومجلس الأخلاقيات في جامعة نيو برنزويك عبر الهاتف : [506-648-5994] أو عبرالبريد الالكتروني : [REB@unb.ca]

أو مجلس الأخلاقيات البحثية لشبكة هورايزون الصحيه عبر الهاتف: [506-648-6094] أو عبر البريد الالكتروني: [REBOffice@HorizonNB.ca]

بيان المشاركين:

لقد قرأت المعلومات حول هذه الدراسة وأتيحت لي الفرصة لمناقشة هذه الدراسة وتم الرد على أسئلتي بما يرضيني.

تم إبلاغي بأن مشاركتي تطوعية وأن البيانات التي أقدمها ستظل سرية. أقر بأنني أوافق بموجبه على المشاركة في هذه الدراسة

اسم المشاركين : ____

توقيع المشاركين:____________________ التاريخ: ____________________

أو

تم الحصول على الموافقة الشفوية من المشارك:____________ التاريخ: ____________________

تاريخ:

يرجى ملاحظة أنه بالموافقة على المشاركة في هذه الدراسة ، فإنك لم تتنازل عن أي حقوق في الرجوع القانوني في حالة

حدوث ضرر متعلق بالبحث.

تصريح من قبل الشخص الذي يقدم معلومات عن الدراسة

لقد شرحت للمشارك المذكور أعلاه طبيعة ومتطلبات والغرض من الدراسة والفوائد المحتملة والمخاطر المحتملة المتعلقه بالمشاركة في هذه الدراسة. لقد أجبت على أي أسئلة تم طرحها. أعتقد أن المشارك يفهم الآثار والطبيعة التطوعية المتعلقة بالدراسة

توقيع الباحث:____________________ التاريخ: ____________________

فريق البحث:

دكتور دانكن ويبستر (Dr. Duncan Webster) قسم الأمراض المعدية ، قسم الطب ، مستشفى سانت جون الإقليمي.

duncan.webster@horizonnb.ca

دكتور إيسادور شولا شامبودا (Isdore Chola Shamputa)

قسم التمريض والعلوم الصحية ، جامعه نيوبرونزويك سانت جون

Chola.Shamputa@unb.ca

دكتور كيمبيرلي باركر (Kimberly Barker) المسؤول الطبي للصحة ، المنطقة الجنوبية ، إدارة الصحة في نيو برونزويك

Kimberley.Barker@gnb.ca

دكتور زوين نوبين (Duyen Nguyen)

كبير مستشاري البرامج الإقليمية، حكومة نيو برونزويك

Duyen.nguyen@gnb.ca

附录 7a

参与者知情同意书

项目名称：优化新不伦瑞克南部新移民的结核病筛查（试点研究）

介绍

我们邀请 19 岁及以上的成年人参与一项关于潜伏（休眠）结核病感染筛查的研究。 本研究的目的是收集信息，以评估为来自有许多结核病病例的国家的新移民实施潜伏性结核病感染筛查计划的可行性。

如果选择参与，您将需要提供大约 4 毫升的血液用于检测潜伏性结核病感染，之后血样将按照圣约翰地区医院的规定进行处理（即不会以任何其他方式储存或使用）。您还需要完成大约 10 分钟的在线调查，以了解您参与研究的整体体验。

您的参与是自愿的，您可以随时退出研究，不会对您在社区内任何相关的其他事物或服务产生任何影响（比如护理、医疗、新移民服务等）。参与研究无需任何费用。参与者将获得 40 加元的电子礼品卡，采血后获得 20 加元，完成在线调查后获得 20 加元。

潜在的好处

参与本研究的潜在好处可能包括确保您没有潜伏的结核病感染（如果检测结果为阴性）。如果测试结果呈阳性而获得了相关的治疗，您将减少患结核病的机会，从而保护您的家人和朋友的安全。

潜在风险：

在采血过程中，采血部位会出现不适、瘀伤或感染，类似于您为其他医学检查提供血样。

隐私和保密

保护您的隐私是此项研究的重要组成部分。从这项研究中收集的信息是严格保密的，您的信息将被匿名化。所有电子数据都将存储在新不伦瑞克大学护理与健康科学系的安全驱动器上以便信息保存和团队内部分析，5 年后销毁。数据仅有研究团队的成员可访问。

问题

如果您在阅读此同意书后如有任何疑问，请询问协助您的研究团队成员。在确定您理解此研究之前，不要口头同意或签署同意书。

该项目已经通过新不伦瑞克大学研究伦理委员会的审查并存档，REB 文件号#033-2021， 并且被 Horizon Health Network 研究伦理委员会认定为最小风险。

如果出现受伤、疾病或残疾，您认为与参与此项研究有关，或者您对此项研究参与者的权利有任何疑问，可以通过电话[506-648-5994]或电子邮件 [REB@unb.ca] 联系新不伦瑞克大学研究主席 Beth Keyes 博士。也可以联系 Horizon Health Network 研究伦理委员会--伦理服务区域总监，联系电话[506) 648-6094]，电子邮件[REBOffice@HorizonNB.ca]。

参与者声明

我已经阅读了有关此项研究的信息，并有机会讨论此项研究，我的问题也得到了满意的回答。我确认我已被告知我参与此项研究是自愿的，我提供的数据保密。我同意参加此项研究。

参与者姓名________________________

参与者签名________________________ 日期____________________

或者

获得参与者的口头同意 ☐ 日期____________________

请注意同意参与此项研究，并不意味您放弃了在发生与此项研究相关的伤害事件时的任何法律追索权。

提供研究信息的人员的声明

我已向上述参与者解释了此项研究的性质、要求、目的、潜在益处以及与参与此项研究相关的可能风险，我已经回答了参与者提出的所有问题。我相信参与者已理解且自愿参与此项研究。

研究员签名：________________________ 日期：____________

研究团队：

Duncan Webster 博士，圣约翰地区医院内科传染病科，duncan.webster@horizonnb.ca

Isdore Chola Shamputa 博士，新不伦瑞克大学圣约翰分校护理与健康科学系，chola.shamputa@unb.ca

Kimberly Barker 博士，新不伦瑞克省卫生部南部地区卫生医疗官，Kimberley.Barker@gnb.ca

Duyen Nguyen 博士，新不伦瑞克省政府区域高级项目顾问，duyen.nguyen@gnb.ca

ضمیمه 7 الف

فرم رضایت آگاهانه برای شرکت کنندگان

عنوان پروژه: بهینه سازی غربالگری سل (توبرکلوز) برای افراد تازه وارد در نیوبرانزویک جنوبی: یک مطالعه آزمایشی

معرفی

ما از بزرگسالان 19 ساله و بزرگتر دعوت می کنیم تا در یک ریسرچ در زمینه غربالگری عفونت سل نهفته (توبرکلوز پنهان) شرکت کنند. هدف از این مطالعه جمع آوری اطلاعات برای ارزیابی امکان اجرای برنامه غربالگری عفونت توبرکلوز پنهان برای تازه واردان از کشورهایی که دارای موارد بالای توبرکلوز هستند.

در صورت انتخاب مشارکت ، از شما خواسته می شود که حدود 4 میلی لیتر از خون خود را جهت تست عفونت توبرکلوزپنهان تهیه کنید که این نمونه خون پس از آزمایش مطابق پروتکل های بیمارستان منطقه ای سنت جان از بین برده میشود. (به عنوان مثال ، ذخیره یا استفاده نمی شود) همچنین از شما خواسته می شود که یک نظرسنجی آنلاین را که تقریبا 10 دقیقه را دربرمیگیرد در مورد تجربه خود از شرکت در این ریسرچ را تکمیل کنید.

مشارکت شما داوطلبانه است و شما می توانید در هر زمان بدون هیچ گونه تأثیری بر مراقبت یا خدمات و یا هر چیز دیگری که شامل تازه واردان است بشمول خدمات/مراکز/خدمات صحی و غیره از این ریسرچ خارج شوید. شرکت در این مطالعه هیچ هزینه ای ندارد. شرکت کنندگان حداکثر 40 دالر کارت تحفه الکترونیکی(e-gift cards) برای وقتی که صرف کرده اند، دریافت می کنند یعنی 20 دالر بعد از اخذ نمونه خون و 20 دالر دیگر بعد از تکمیل نظرسنجی آنلاین/ ریسرچ

مزایای احتمالی

مزایای احتمالی شرکت در این ریسرچ ممکن است شامل اطمینان خاطر از عدم ابتلا به توبرکلو (در صورت منفی بودن تست) باشد. اگر تست مثبت باشد و تحت درمان قرار بگیرید ، چانس ابتلا به بیماری توبرکلوز کاهش چشمگیر یافته ، و در نتیجه خانواده و دوستان خود را مصون نگه می دارید.

خطرات احتمالی:

به میزان کمی خطر ناراحتی ، کبودی یا عفونت در قسمت اخذ نمونه خون وجود دارد ، که این مشابه اخذ خون برای هر تست خون می باشد.

حریم شخصی و محرمانه بودن

حفاظت از حریم شخصی شما بخش مهمی از این ریسرچ است. معلومات جمع آوری شده از این ریسرچ کاملاً محرمانه است و معلومات شما ناشناس خواهد بود. تمام معلومات الکترونیکی/ کامپیوتری به مدت 5 سال در یک درایو امن در بخش نرسنگ و علوم صحی دانشگاه نیوبرانزویک ذخیره می شوند تا امکان انتشار معلومات فراهم شود و بعد از آن از بین می رود. معلومات و آمار فقط توسط اعضای تیم ریسرچ قابل دسترسی است.

سوالات

اگر بعد از خواندن این فرم سوالاتی داشتید، از اعضای تیم تحقیقاتی که شما را ارزیابی می کنند، بپرسید. تا زمانی که مطمئن نشده اید که ریسرچ را متوجه شده اید ، نباید این فرمه را امضا کنید یا رضایت شفاهی ارائه دهید.

این پروژه توسط هیئت بررسی کننده اصول اخلاقی ریسرچ دانشگاه نیوبرانزویک که به عنوان REB #033-2021 درفایل است، و هیئت اخلاق تحقیقاتی شبکه صحی هورایزن (Horizon) مورد بررسی قرار گرفته است و حداقل خطر را به همراه دارد. در صورت وارد شدن صدمه، بیماری یا معلولیت که معتقد هستید این امر مربوط به مشارکت شما در این ریسرچ است ، یا

در صورت داشتن هرگونه سوالات در مورد حقوق خود به عنوان یک شرکت کننده در تحقیق ، می توانید با دکتر بیت گیز ، رئیس هیئت بررسی کننده اصول اخلاقی ریسرچ در UNBSJ با تلفن [5994-648-5994] یا از طریق ایمیل [REB@unb.ca] تماس بگیرید یا مدیر منطقه ای خدمات اصول اخلاقی ، هیئت اصول اخلاق ریسرچ شبکه صحی Horizon با تلفن [6094-648(506)] یا از طریق ایمیل به آدرس [REBOffice@HorizonNB.ca].

بیانیه شرکت کنندگان

من معلومات مربوط به این ریسرچ را خوانده ام و فرصت برای بحث در مورد این ریسرچ به من داده شد و در قبال سوالات خود پاسخ قناعت بخش دریافت کردم. تصدیق می کنم که به من اطلاع داده شده است که مشارکت من داوطلبانه است و معلوماتی که ارائه می دهم محرمانه خواهد ماند. بدینوسیله با شرکت در این ریسرچ موافقت می کنم.

نام شرکت کننده ____________________

امضای شرکت کننده ____________________ تاریخ ____________________

یا

اخذ رضایت شفاهی شرکت کننده ☐ تاریخ ____________________

لطفاً توجه داشته باشید که با موافقت به شرکت در این ریسرچ، از هیچگونه حق توسل به قانون در صورت صدمه ناشی از تحقیق/ ریسرچ چشم پوشی نکرده اید.

اظهارنظر توسط شخص ارائه کننده اطلاعات در مورد مطالعه

من ماهیت ، الزامات و هدف ریسرچ، مزایای احتمالی و خطرات احتمالی مرتبط با شرکت در این ریسرچ را برای شرکت کننده فوق توضیح دادم. من به هر سوالی که مطرح شد، پاسخ داده ام. من معتقدم که شرکت کننده مفاهیم و ماهیت داوطلبانه ریسرچ را درک کرده است.

امضای ریسرچ کننده: ____________________ تاریخ: ____________________

گروه تحقیق:

دکتر دانکن وبستر ، بخش بیماریهای عفونی ، گروه پزشکی ، بیمارستان منطقه ای سنت جان ، duncan.webster@horizonnb.ca

دکتر ایزدور چولا شمپوتا ، گروه نرسنگ و علوم صحی ، دانشگاه نیوبرانزویک سنت جان ، chola.shamputa@unb.ca

دکتر کیمبرلی بارکر ، افسر پزشکی صحت ، منطقه جنوبی ، وزارت صحت نیوبرانزویک ، Kimberley.Barker@gnb.ca

دکتر دوین نگوین ، مشاور ارشد برنامه منطقه ای ، دولت نیوبرانزویک ، duyen.nguyen@gnb.ca
