# Supplementary material for: Optimizing tuberculosis screening for immigrants in southern New Brunswick: A pilot study protocol
Source: PLoS One. 2022 Nov 4;17(11):e0277255. doi: 10.1371/journal.pone.0277255 (PMC9635694; doi:10.1371/journal.pone.0277255)
Supplement: S2 Appendix — (PDF) [file pone.0277255.s002.pdf]

Appendix 2

Participant Survey

Instructions: Please circle the response that best describes your agreement with the statement.

1. I received information on why the latent tuberculosis test was being done.

Strongly Disagree	Disagree	Neutral	Agree	Strongly Agree
1	2	3	4	5

2. The blood collection office was easy to find.

Strongly Disagree	Disagree	Neutral	Agree	Strongly Agree
1	2	3	4	5

3. The blood collection office was easy to travel to.

Strongly Disagree	Disagree	Neutral	Agree	Strongly Agree
1	2	3	4	5

4. The blood collection process was simple (e.g. registration, blood collection).

Strongly Disagree	Disagree	Neutral	Agree	Strongly Agree
1	2	3	4	5

5. The waiting time for blood collection was reasonable.

Too long	Somewhat long	Neutral	Somewhat short	Very short
1	2	3	4	5

6. The healthcare provider (i.e., doctor, nurse) answered all my questions well.

Strongly Disagree	Disagree	Neutral	Agree	Strongly Agree
1	2	3	4	5

7. I was satisfied with the overall experience with the latent tuberculosis screening process and/or care I received.

Strongly Disagree	Disagree	Neutral	Agree	Strongly Agree
1	2	3	4	5

If you were not satisfied, why?

8. I would recommend other people to do a latent tuberculosis screening test.

Strongly Disagree	Disagree	Neutral	Agree	Strongly Agree
1	2	3	4	5

If you not, please explain: correct this

9. My knowledge regarding tuberculosis improved by participating in the study.

Strongly Disagree	Disagree	Neutral	Agree	Strongly Agree
1	2	3	4	5

Please explain:

10. My attitudes regarding tuberculosis improved by participating in the study.

Strongly Disagree	Disagree	Neutral	Agree	Strongly Agree
1	2	3	4	5

Please explain:

11. Do you have any suggestions on how the latent tuberculosis testing process can be improved?

Yes	No
1	2

If yes please provide your suggestions below:

Annexe 2
Sondage auprès des participants

Instructions : Veuillez encercler la réponse qui décrit le mieux votre accord avec l'énoncé.

1. J'ai reçu des informations sur la raison pour laquelle le test de tuberculose latente était effectué.

Pas du tout d'accord --Pas d'accord-- Neutre --D'accord --Tout à fait d'accord
1 2 3 4 5

2. Le bureau de prélèvement sanguin était facile à trouver.

Pas du tout d'accord --Pas d'accord –Neutre-- D'accord --Tout à fait d'accord
1 2 3 4 5

3. Le bureau de prélèvement sanguin était facile d'accès.

Pas du tout d'accord-- Pas d'accord-- Neutre --D'accord --Tout à fait d'accord
1 2 3 4 5

4. Le processus de collecte de sang était simple (par exemple, enregistrement, collecte de sang).

Pas du tout d'accord-- Pas d'accord-- Neutre ---D'accord-- Tout à fait d'accord
1 2 3 4 5

5. Le temps d'attente pour le prélèvement sanguin était raisonnable.

Trop long --Un peu long –Neutre-- Un peu court-- Très court
1 2 3 4 5

6. Le fournisseur de soins de santé (c'est-à-dire le médecin, l'infirmière) a bien répondu à toutes mes questions.

Pas du tout d'accord-- Pas d'accord –Neutre-- D'accord--- Tout à fait d'accord
1 2 3 4 5

7. J'ai été satisfait de l'expérience globale du processus de dépistage de la tuberculose latente et/ou des soins que j'ai reçus.

Pas du tout d'accord-- Pas d'accord-- Neutre ---D'accord ---Tout à fait d'accord
1. 2 3 4 5

**Si vous n'étiez pas satisfait, pourquoi ?**

8. Je recommanderais à d'autres personnes de faire un test de dépistage de la tuberculose latente.

Pas du tout d'accord --Pas d'accord—Neutre-- D'accord --Tout à fait d'accord
1 2 3 4 5

**Si ce n'est pas le cas, veuillez expliquer : corriger ceci**

9. Mes connaissances sur la tuberculose se sont améliorées en participant à l'étude.

Pas du tout d'accord --Pas d'accord –Neutre-- D'accord-- Tout à fait d'accord
1 2 3 4 5

**Veuillez expliquer :**

10. Mes attitudes vis-à-vis de la tuberculose se sont améliorées en participant à l'étude.

Pas du tout d'accord--	Pas d'accord--	Neutre ---	D'accord ---	Tout à fait d'accord
1	2	3	4	5

Veuillez expliquer :

11. Avez-vous des suggestions sur la manière d'améliorer le processus de dépistage de la tuberculose latente ?

Oui	Non
1	2

Si oui, veuillez fournir votre suggestion ci-dessous :

Appendix 2

Encuesta de participantes

Instrucciones: Favor de indicar la respuesta con que usted está de acuerdo.

1. Recibí información acerca del por qué esta investigación de tuberculosis latente fue realizada.

Estoy muy en desacuerdo	No estoy de acuerdo	Neutral	Estoy de acuerdo	Estoy muy de acuerdo
1	2	3	4	5

2. La oficina de colección de sangre fue fácil encontrar.

Strongly Disagree	Disagree	Neutral	Agree	Strongly Agree
1	2	3	4	5

3. Fue fácil viajar a la oficina de colección de sangre.

Strongly Disagree	Disagree	Neutral	Agree	Strongly Agree
1	2	3	4	5

4. El proceso de recoger sangre fue simple (ej. Registración, colección de sangre).

Strongly Disagree	Disagree	Neutral	Agree	Strongly Agree
1	2	3	4	5

5. El tiempo de espera para la colección de sangre no era demasiado largo.

Too long	Somewhat long	Neutral	Somewhat short	Very short
1	2	3	4	5

6. El/la profesional de salud (ej. Médico, enfermera) respondió a todas mis preguntas.

Strongly Disagree	Disagree	Neutral	Agree	Strongly Agree
1	2	3	4	5

7. Estaba muy satisfecho con la experiencia del proceso de la prueba de tuberculosis latente en general y/o la atención que recibí.

Strongly Disagree	Disagree	Neutral	Agree	Strongly Agree
1	2	3	4	5

Sí usted no estaba satisfecho con la experiencia, díganos por qué.

8. Yo recomendaría a otras personas hacer la prueba de latente tuberculosis.

Strongly Disagree	Disagree	Neutral	Agree	Strongly Agree
1	2	3	4	5

Si no recomendaría esta experiencia a otras personas, por favor explíca sus razones.

9. Mi conocimiento acerca de tuberculosis mejoró por participar en esta investigación.

Strongly Disagree	Disagree	Neutral	Agree	Strongly Agree
1	2	3	4	5

Favor de explicar por qué o por qué no

10. Mis actitudes acerca de tuberculosis mejoraron por participar en esta investigación.

Strongly Disagree	Disagree	Neutral	Agree	Strongly Agree
1	2	3	4	5

Favor de explicar por qué o por qué no

11. Tiene usted alguna sugerencia en como podemos mejorar el proceso de realizar la prueba de tuberculosis latente?

Sí	No
1	2

Si respondió "sí" a esta pregunta, favor de contarnos sus sugerencias.

Appendix 2

Sahanka Ka-qaybgalaha

Tilmaamaha: Fadlan goobaabin jawaabta sida wanaagsan u qeexaysa heshiiskaaga bayaanka.

1. Waxaan helay xog ku saabsan sababta baaritaanka qaaxada qarsoon loo sameeyey.

Aad u Diidane	Diidan	Dhexdhexaad	Ogol	Aad Ayaan U Ogolahay
1	2	3	4	5

2. Xafiiska ururinta dhiigga ayaa ahaa mid sahlan in la helo.

Aad u Diidanee	Diidan	Dhexdhexaad	Ogol	Aad Ayaan U Ogolahay
1	2	3	4	5

3. Xafiiska dhiiga ayaa ahaa mid sahlan in loo safro.

Aad u Diidaneee	Diidan	Dhexdhexaad	Ogo	Aad Ayaan U Ogolahay
1	2	3	4	5

4. Habka ururinta dhiiggu wuxuu ahaa mid sahlan (tusaale, diiwaangelin, ururinta dhiigga).

Aad u Diidane	Diidan	Dhexdhexaad	Ogol	Aad Ayaan U Ogolahay
1	2	3	4	5

5. Waqtiga sugitaanka ururinta dhiiggu waa macquul.

Aad u dheer	Waxoogaa dheer	Dhexdhexaad	Waxoogaa gaaban	Aad u gaaban
1	2	3	4	5

6. Bixiyaha daryeelka caafimaadka (tusaale ahaan, dhakhtar, kalkaaliso) ayaa si fiican iiga jawaabay dhammaan su'aalahaygii.

Aad u Diidane	Diidan	Dhexdhexaad	Ogol	Aad Ayaan U Ogolahay
1	2	3	4	5

7. Waxaan ku qancay waayo -aragnimada guud ee habka baaritaanka qaaxada qarsoon iyo/ama daryeelka aan helay.

Aad u Diidane	Diidan	Dhexdhexaad	Ogol	Aad Ayaan U Ogolahaye
1	2	3	4	5

Haddii aadan ku qanacsanayn, sababta?

8. Waxaan kula talin lahaa dadka kale inay sameeyaan baaritaanka baarista qaaxada ee qarsoon.

haddii aadan samayn, fadlan sharrax tan saxan

Aad u Diidane	Diidan	Dhexdhexaad	Ogol	Aad Ayaan U Ogolahay
1	2	3	4	5

9. Aqoonta aan u leeyahay qaaxada ayaa hagaagtay ka -qaybgalka daraasadda.

Fadlan sharax:

Aad u Diidane	Diidan	Dhexdhexaad	Ogol	Aad Ayaan U Ogolahay
1	2	3	4	5

10. Dabcigayga ku aaddan qaaxada ayaa wanaajiyay ka -qaybgalka daraasadda.

Fadlan sharax:

Aad u Diidane	Diidan	Dhexdhexaad	Ogol	Aad Ayaan U Ogolahay
1	2	3	4	5

11. Ma haysaa wax talo ah oo ku saabsan sida habka baaritaanka qaaxada qarsoon loo horumarin karo?

Haa	Maya
1	2

If yes please provide your suggestions below:

2

استبيان المشاركين

التعليمات: يرجي وضع دائرة حول الرد الذي يصف موافقتك على البيان على أفضل وجه.

1. لقد تلقيت معلومات عن سبب إجراء اختبار السل الكامن.

لا اوافق بشدة	لا اوافق	محايد	اوافق	اوافق بشدة
1	2	3	4	5

2. لقد كان من السهل ايجاد مكتب جمع عينات الدم.

لا اوافق بشدة	لا اوافق	محايد	اوافق	اوافق بشدة
1	2	3	4	5

3. لقد كان من السهل الوصول الي مكتب جمع عينات الدم.

لا اوافق بشدة	لا اوافق	محايد	اوافق	اوافق بشدة
1	2	3	4	5

4. لقد كانت إجراءات عملية جمع عينات الدم بسيطة (مثل التسجيل ، جمع عينات الدم).

لا اوافق بشدة	لا اوافق	محايد	اوافق	اوافق بشدة
1	2	3	4	5

5. لقد كان وقت الانتظار لسحب عينة الدم معقولاً.

طويل جدا	طويل نوعا ما	معقول	قصير	قصيرجداً
1	2	3	4	5

6. لقد أجاب مقدم الرعاية الصحية (أي الطبيب والممرضة) على جميع أسئلتي بشكل جيد.

لا اوافق بشدة	لا اوافق	محايد	اوافق	اوافق بشدة
1	2	3	4	5

7. لقد كنت راضيًا بشكل عام عن عملية فحص السل الكامن و / أو الرعاية التي تلقيتها.

لا اوافق بشدة	لا اوافق	محايد	اوافق	اوافق بشدة
1	2	3	4	5

إذا لم تكن راضيًا ، لماذا؟

8. أرغب في أن أوصي الآخرين بإجراء اختبار فحص السل الكامن.

لا اوافق بشدة	لا اوافق	محايد	اوافق	اوافق بشدة
1	2	3	4	5

إذا كنت لا توصي بذلك ، يرجى التوضيح: صحح هذا

9. لقد تحسنت معرفتي بمرض السل من خلال مشاركتي في هذه الدراسة.

لا اوافق بشدة	لا اوافق	محايد	اوافق	اوافق بشدة
1	2	3	4	5

يرجى التوضيح:

10. لقد تحسن موقفي تجاه مرض السل من خلال مشاركتي في هذه الدراسة.

لا اوافق بشدة	لا اوافق	محايد	اوافق	اوافق بشدة
1	2	3	4	5

يرجى التوضيح:

11. هل لديك أي اقتراحات حول كيفية تحسين عملية اختبار السل الكامن؟

نعم	لا
1	2

إذا كانت الإجابة بنعم ، يرجى تقديم اقتراحاتك أدناه:

附录 2
参与者调查

说明：请圈出最能描述您观点和意愿的答案。

1. 我收到了关于为什么要进行潜伏性结核病检测的信息。

非常不同意	不同意	中立	同意	非常同意
1	2	3	4	5

2. 血样采集中心很容易找到。

非常不同意	不同意	中立	同意	非常同意
1	2	3	4	5

3. 血样采集中心交通便利。

非常不同意	不同意	中立	同意	非常同意
1	2	3	4	5

4. 血样采集过程简单（比如登记、血样采集）。

非常不同意	不同意	中立	同意	非常同意
1	2	3	4	5

5. 血样采集等待时间合理。

时间太长	时间有点长	中立	时间比较短	时间很短
1	2	3	4	5

6. 医疗保健提供者（即医生、护士）很好的回答了我所有的问题。

非常不同意	不同意	中立	同意	非常同意
1	2	3	4	5

7. 我对此次的潜伏性结核病筛查过程和我接受的整体护理体验感到满意。

非常不同意	不同意	中立	同意	非常同意
1	2	3	4	5

如果您不满意，为什么？

8. 我会推荐其他人做潜伏性结核病筛查测试。

非常不同意	不同意	中立	同意	非常同意
1	2	3	4	5

如果您不同意，请解释说明：

9. 通过参加此次研究提高了我对结核病的认识。

非常不同意	不同意	中立	同意	非常同意
1	2	3	4	5

请解释说明：

10. 通过参与这项研究，我对结核病的态度有所改善。

非常不同意	不同意	中立	同意	非常同意
1	2	3	4	5

请解释说明：

11. 您对如何改进潜伏性结核检测过程有什么建议吗？

有	没有
1	2

如果有，请将您的建议写在下面

نظرسنجی شرکت کننده

دستورالعمل: لطفاً پاسخی را حلقه کنید که به بهترین نحو نظرشما را توصیف می کند.

1. من معلومات در مورد علت انجام تست توبرکلوز پنهان دریافت کردم.

کاملاً مخالف	موافق نیستم	بدون نظر	موافقم	کاملاً موافقم
1	2	3	4	5

2. آفیس/اداره جمع آوری نمونه خون به راحتی یافت شد.

کاملاً مخالف	موافق نیستم	بدون نظر	موافقم	کاملاً موافقم
1	2	3	4	5

3. مسیر پیمودن به افیس جمع آوری نمونه خون آسان بود.

کاملاً مخالف	موافق نیستم	بدون نظر	موافقم	کاملاً موافقم
1	2	3	4	5

4. پروسه جمع آوری نمونه خون ساده بود (به عنوان مثال ثبت ، جمع آوری خون).

کاملاً مخالف	موافق نیستم	بدون نظر	موافقم	کاملاً موافقم
1	2	3	4	5

5. زمان انتظار برای جمع آوری خون معقول بود.

کاملاً مخالف	موافق نیستم	بدون نظر	موافقم	کاملاً موافقم
1	2	3	4	5

6. عرضه کننده مراقبت های صحی (یعنی داکتر ، نرس) به تمام سوالات من به خوبی پاسخ داد.

کاملاً مخالف	موافق نیستم	بدون نظر	موافقم	کاملاً موافقم
1	2	3	4	5

7. من از تجربه کلی درمورد مراحل غربالگری/ سکرینگ توبرکلوز و/یا مراقبت دریافتی راضی بودم.

کاملاً مخالف	موافق نیستم	بدون نظر	موافقم	کاملاً موافقم
1	2	3	4	5

8. من به افراد دیگر توصیه می کنم تست غربالگری/سکرینینگ توبرکلوز پنهان را انجام دهند.

کاملاً مخالف	موافق نیستم	بدون نظر	موافقم	کاملاً موافقم
1	2	3	4	5

9. دانش من در مورد توبرکلوز با شرکت در ریسرچ بهبود یافت.

کاملاً مخالف	موافق نیستم	بدون نظر	موافقم	کاملاً موافقم
1	2	3	4	5

10. طرز برخورد و رفتار من در مورد توبرکلوز با شرکت در این ریسرچ بهبود یافت.

کاملاً مخالف	موافق نیستم	بدون نظر	موافقم	کاملاً موافقم
1	2	3	4	5

11. آیا پیشنهادی در مورد نحوه بهبود پروسه تست توبرکلوز پنهان دارید؟

کاملاً مخالف	موافق نیستم	بدون نظر	موافقم	کاملاً موافقم
1	2	3	4	5
