# Supplementary material for: Optimizing tuberculosis screening for immigrants in southern New Brunswick: A pilot study protocol
Source: PLoS One. 2022 Nov 4;17(11):e0277255. doi: 10.1371/journal.pone.0277255 (PMC9635694; doi:10.1371/journal.pone.0277255)
Supplement: S3 Appendix — (PDF) [file pone.0277255.s003.pdf]

Appendix 1:
LTBI Indicator Form

Patient ID (assigned by researcher):___________________________

Patient Demographic Information:

1. Date of birth________________________

2. Sex: 1. Male 2. Female

3. Gender: 1. Male 2. Female 3. Other: ________________

4. Country/Region of birth: ________________

5. Countries where you lived for more than 6 months (before coming to Canada):_________

   ____

   ____

   ____

6. Date of immigration/arrival to Canada: _____________________

7. Immigration stream:

   1. Economic 2. Family Reunification 3. Study Permit

   4. Work Permit 5. Refugee/Refugee Claimant 6. Temporary Visitors

Pre-Visit Data: (to be completed by Public Health)

8. New LTBI case? 1. Yes 2. No

9. Known prior contact with active TB? 1.Yes 2. No 3. Unknown; If yes, when?________

10. Client enrolled in IGRA study? 1. Yes 2. No

11. Medical Surveillance client?. 1. Yes 2. No

12. TST…………………………………..__________________(mm of induration)

13. IGRA……………………………………1. Positive 2. Negative 3. Not performed

14. IGRA result data…………….. TB1:________; TB2:_________; NIL:__________

15. Date of referral for LTBI treatment assessment:______________________________

<u>Visit Data:</u> (to be completed by assessing healthcare provider)

16. Referred LTBI patient seen within 30 days for treatment assessment. 1. Yes 2. No

17. Referred LTBI Patient seen within 90 days for treatment assessment.

    1. Yes 2. No 3. N/A

18. Referral source____________________Public Health / ID / Other: ________________

19. BCG vaccine 1. Yes 2. No 3. Unknown

20. Presence of comorbidities increasing risk for activation. 1. Yes 2. No

21. TST in 3D score……….% risk for active TB within next 2 years / % risk for active TB before age 80 years

22. Treatment 1. Yes 2. Prior adequate treatment 3. Refused

23. Planned treatment start date: ____

24. Drug regimen chosen or previously administered: _____________________________

<u>Post-Visit Data:</u> (to be completed by assessing healthcare provider)

25. Completed treatment: 1. Yes 2. No

26. # missed doses: ____

27. Adverse effects reported: 1. Yes 2. No

    Clinically significant ALT rise (>2-3x upper limit normal?). 1. Yes 2. No

28. 80% or greater compliance with risk adjusted follow up schedule: 1. Yes 2. No

29. Agrees to annual follow up CXR x 2 years. 1. Yes 2. No 3. N/A
