# Supplementary material for: Optimizing tuberculosis screening for immigrants in southern New Brunswick: A pilot study protocol
Source: PLoS One. 2022 Nov 4;17(11):e0277255. doi: 10.1371/journal.pone.0277255 (PMC9635694; doi:10.1371/journal.pone.0277255)
Supplement: S4 Appendix — (PDF) [file pone.0277255.s004.pdf]

Appendix 3

Health Care Practitioner Survey

1. What was your role in this study? 1. NP 2. ID Physician

2. Did you feel the patients were appropriately supported? 1. Yes 2. No

**Please explain:**

3. Were there any barriers in supporting the patients during the initial visit?

   1. Yes 2 No

**If yes, what were the barriers and how could they have been prevented?**

4. Were there any barriers in supporting the patients during the subsequent visit?

   1. Yes 2 . No

**If yes, what were the barriers and how could they have been prevented?**

5. Did you feel you had all the tools to care for the patients in your role?

   1. Yes 2. No

**If no, what tools were missing?**

6. Were there any barriers in arranging follow up tests or visits?

   1. Yes 2. No

If yes, what were the barriers and how could they have been prevented?

7. What was helpful in caring for the patients?

Please state them bleow:

8. On average how long did you spend with each patient per visit (in hours)?

Please state them bleow:

9. How much time did you spend on patient management in total including administration, diagnostics, & arrangements?

Please state them bleow:

10. Do you have any final comments 1. Yes 2. No

If yes, please state them bleow:
