# Supplementary material for: Optimizing tuberculosis screening for immigrants in southern New Brunswick: A pilot study protocol
Source: PLoS One. 2022 Nov 4;17(11):e0277255. doi: 10.1371/journal.pone.0277255 (PMC9635694; doi:10.1371/journal.pone.0277255)
Supplement: Attachment — Submitted filename: Response to Reviewers comments.docx [file pone.0277255.s006.docx]

**Optimizing tuberculosis screening for immigrants in southern New Brunswick: A pilot study Protocol**

Responses to Reviewers’ comments:

Below are our responses to the reviewers’ comments:

(1) Please, define the abbreviations if it used first in the manuscript body. (HCP, NB, PC)
For example, full name of 'HCP' is missing in both abstract and manuscript body.
**Response**: i) “HCP” has been defined in line 36; ii) NB and PC are addressed below:

(2) Please, minimize the use of the abbreviation.
For example, I think that it is unnecessary to use "NB" in the abstract, because it is used only one time in the abstract. Another example is "PC".

**Response**: i) “NB” abbreviation has been deleted from the abstract and introduced in the manuscript body (see line 81) and ii)“PC”, this abbreviation has been deleted and replaced by “Personal Computers.” (see lines 257 and 258).

(3) I think you need to insert brackets before and after "SGBA+".

**Response**: brackets have been inserted (see line 233).

(4) Please, check any minor errors. PLOS ONE does not copyedit accepted manuscripts.
**Response**: This has been done.

Reviewer #2, “...I note that the authors comment that data will be presented in their final form in aggregated form, however I encourage the authors to continue with plans to analyze AND present the data by sub-tabulations as possible (e.g., by sex) so that the results can be most useful to other researchers and LTBI program managers.”

**Response**: This will be done.
