# Supplementary material for: Optimizing tuberculosis screening for immigrants in southern New Brunswick: A pilot study protocol
Source: PLoS One. 2022 Nov 4;17(11):e0277255. doi: 10.1371/journal.pone.0277255 (PMC9635694; doi:10.1371/journal.pone.0277255)
Supplement: Attachment — Submitted filename: Responses to Reviewers.docx [file pone.0277255.s007.docx]

Responses to Reviewers' comments

| **Editor’s comments** | **Author Response** |
| --- | --- |
| (1) Define abbreviations upon first appearance in the text. : IGRA is not defined in the manuscript body. | IGRA has been defined in the manuscript (see line 94). |
| (2) Keep abbreviations to a minimum. : 'COVID-19' is only used once. Please, change the "COVID-19" to "coronavirus disease 2019". | As suggested, COVID-19" has now been changed to "coronavirus disease 2019" (see line 194). |
| (3) The author states that "This sutdy uses the fourth-generaion IGRA". I thick this assays is the QuantiFERON-TB Gold Plus. Please, mention the name of the assay and its manufacturer in an appropirate place. | That is correct. The statement in question has been updated and now reads as follows,  “This study is using the QuantiFERON-TB Gold Plus (QFT-Plus) interferon-gamma release assay (IGRA) (QIAGEN),....” (see lines 93-94). |
| (4) Please, check any minor erros. PLOS ONE does not copyedit accepted manuscripts. | This has been done. |
